# Chromium/Vanadium Mixed Oxide Films on Pt(111): Revealing Oxide Alloying Mechanisms in Two Dimensions

**DOI:** 10.1021/acsami.5c05932

**Published:** 2025-07-25

**Authors:** Ghada Missaoui, Piotr Igor Wemhoff, Jacek Goniakowski, Claudine Noguera, Niklas Nilius

**Affiliations:** † Institut für Physik, 232751 Carl von Ossietzky Universität Oldenburg, D-26111 Oldenburg, Germany; ‡ Institut des Nanosciences de Paris, UMR 7588, 84219 CNRS−Sorbonne Université, F-75005 Paris, France

**Keywords:** oxide alloying, 2D oxide systems, chromium, vanadium, scanning tunneling microscopy, density functional theory

## Abstract

The mixing characteristics of oxide materials largely depend on the dimensionality of the system, and many oxide-alloy structures in three dimensions (3D) do not have a 2D analog. To unravel fundamental alloying mechanisms in 2D, V/Cr mixing into oxide thin films is investigated on Pt(111) by scanning tunneling microscopy and density functional theory. The experiments reveal flat, double-stack islands made of a compact bottom and a honeycomb top layer with a 4.5 Å total height. The energetically most favorable structure-match comprises an O–Cr–O trilayer at the interface to the Pt(111) capped by a mixed V/Cr honeycomb top layer. The structure is stabilized by strong interlayer adhesion, reinforced by a charge transfer toward the central trilayer from the metal support and the honeycomb plane. A negative V/Cr mixing enthalpy arises from the presence of two distinct surface sites that enable formation of tetrahedrally coordinated V^5+^ and octahedrally coordinated Cr^3+^ cations. The identified thin-film structure bears resemblance to a (111) cut of a hypothetical V/Cr spinel, a unique 2D configuration without bulk equivalent that is stabilized solely by its nanoscale thickness and a strong coupling to the Pt support.

## Graphical abstract

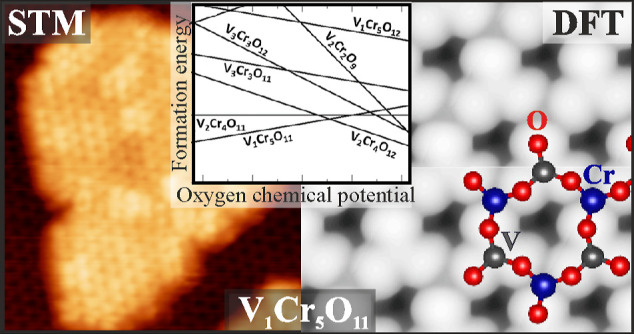

## 1 Introduction

Although alloying has been long recognized as a powerful tool to tailor material properties for various applications, the physics and chemistry behind cationic mixing are largely unexplored for two-dimensional (2D) oxides grown on metal supports. This stands in obvious contrast to the technological relevance of such systems, for instance, in reverse catalysts or materials with strong metal-support interactions.^1,2^ In both cases, the growth of ultrathin oxide layers, often with ternary composition, leads to the formation of active sites for the desired catalytic process. To date, only a limited number of doped and mixed 2D oxides have been experimentally characterized at a true atomic level, typically with scanning tunneling (STM) and atomic force microscopy (AFM).^3–7^ Moreover, density functional theory (DFT) simulations have suggested that the mixing characteristics are largely governed by the nature of the supporting metal.^8,9^ The effect was exemplarily demonstrated for V/Fe mixed oxide films that show a complex alloying behavior, even though no such mixed phases exist in the respective bulk system. More precisely, V_2–*x*_Fe_*x*_O_3_ honeycomb (hc) films can be grown over a wide range of Fe concentrations on Pt(111), stabilized by a strong charge-transfer-mediated coupling of the V ions to the Pt support.^10^ In contrast, V/Fe mixing on Ru(0001) leads to hc-layers with unique VFeO_6_ stoichiometry, as an interfacial oxygen layer prevents charge exchange between oxide and metal support.^11^ Apparently, the nature of 2D oxide alloys can be altered by selecting favorable substrates and oxidation conditions for cationic mixing.^12^

Most of the 2D mixed oxides discussed above consist of a single cationic plane and expose equivalent lattice sites only. Alloying effects are therefore primarily governed by electrostatic forces, both between neighboring cations and the support and much less by structural peculiarities of the lattice. In contrast, 3D mixed oxides, such as silicates^13^ or spinels,^14,15^ always feature inequivalent lattice sites. For example, tetrahedrally and octahedrally coordinated cations alternate in spinel lattices and become populated by high- and low-valence cations, respectively. Therefore, structural preconditions also have a large impact on cationic mixing but were, to our best knowledge, not investigated in 2D mixed oxides so far.

The understanding of oxide alloying in 2D is still in its infancy, as the controlled fabrication and atomic-scale characterization of ternary oxide materials are challenging. One limitation is the inability of AFM and STM techniques to resolve the inner structure of multilayer oxide films. Another difficulty arises from the electron exchange with the metal support, which may obscure the interpretation of the spectroscopic results. In fact, certain alloy configurations, e.g., spinel structures in pure and mixed Cr-based oxides,^16–18^ were assigned solely by their formal stoichiometry measured with photoelectron spectroscopy and not by a true structural analysis. This deficiency is overcome in the present study on V/Cr mixed oxide films on Pt(111), which were characterized by atomic-scale STM imaging, while underlying mixing principles were derived from DFT calculations. Our findings reveal that the films have a double-stack character, comprising an O–Cr–O trilayer with octahedrally coordinated Cr ions at the interface and a hc-bilayer with Cr and V ions in octahedral and tetrahedral sites at the surface. Driving forces for mixing are the strong preference of the cations for specific binding environments and the large electron transfer toward the central trilayer from both the metal support and the surface hc-plane. In addition, we discuss the relationship between the observed film structure, and a hypothetical V/Cr nanospinel that can only be stabilized in the limit of ultrathin films.^19^

## 2 Experimental and Theoretical Methods

All experiments were performed in an ultrahigh vacuum chamber (*p* ∼ 2 × 10^–10^ mbar), equipped with a liquid-nitrogen-cooled STM, an electron-diffraction (LEED) setup, as well as standard tools for thin-film preparation and analysis. The STM measurements were conducted with electrochemically etched Au tips in the constant current mode. The Pt(111) substrate was cleaned by alternating cycles of Ar^+^ sputtering and vacuum annealing at 1250 K until a sharp (1 × 1) spot pattern and large, atomically flat terraces were obtained in LEED and STM, respectively. Cr and V were deposited with a dual electron-beam evaporator calibrated with a quartz microbalance. The samples were oxidized in 1 × 10^–6^ mbar O_2_ at 650 K; film crystallization was realized by either vacuum or O_2_ annealing at 650 K.

DFT calculations were performed with the Vienna Ab-initio Simulation Package (VASP),^20,21^ using the projector augmented wave method to represent electron–core interactions and a 400 eV energy cutoff in developing the Kohn–Sham orbitals on a plane-wave basis set.^22,23^ A dispersion-corrected exchange–correlation functional (optB88-vdW)^24,25^ was employed within the DFT + *U* approach proposed by Dudarev.^26,27^ As in our previous studies,^8,9,28,29^ we utilized *U* values close to those reported in the literature: *U* = 1.7 eV for V and 3.0 eV for Cr in the sesquioxides. All calculations were spin-polarized, and the relative stability of nonmagnetic versus magnetic solutions, with either parallel or antiparallel spin moments, was systematically tested. Ionic charges were estimated with the Bader partition scheme,^30,31^ and magnetic moments were obtained by integrating the spin density within the Bader volumes. The Tersoff–Hamann approximation was used for STM simulations,^32^ and atomic configurations were plotted with VESTA.^33^ Oxide films were deposited on slabs of four Pt(111) planes. Atoms in the lowest Pt plane were frozen at bulk positions, while those in the upper planes could move perpendicular to the surface. The coordinates of all oxide ions were allowed to relax until the forces dropped below 0.01 eV Å^–1^. The formation energies (eV/cation) of mixed (2 × 2) V_*m*_Cr_*n*_O_*k*_/Pt configurations were evaluated as a function of the oxygen chemical potential Δμ_O_ with respect to observed binary references, i.e., (2 × 2) V_2_O_3_/Pt hc-bilayers^10,34^ and (√3×√3)­R30° Cr_3_O_6_/Pt trilayers^35^

$$E_{form}=\frac{1}{m+n}\left[E\left(V_{m}{{Cr}_{n}O}_{k}/Pt\right)-E\left(Pt\right)-\frac{m}{2}\left(E\left({V_{2}O}_{3}/Pt\right)-E\left(Pt\right)\right)-\frac{n}{3}\left(E\left({{Cr}_{3}O}_{6}/Pt\right)-\frac{3}{4}E\left(Pt\right)\right)-\left(k-1.5m-2n\right)\left(\frac{1}{2}E\left(O_{2}\right)+\Delta \mu_{O}\right)\right]$$

Here, *E*(V_*m*_Cr_*n*_O_*k*_/Pt), *E*(V_2_O_3_/Pt), and *E*(Pt) are the total energies of the supported (2 × 2) films and the Pt substrate, *E*(Cr_3_O_6_/Pt) is the total energy of the supported (√3×√3)­R30° film, and *E*(O_2_) refers to the total energy of an oxygen molecule.

## 3 Experimental Results

### 3.1 Binary Parent Oxides

To evaluate the impact of cationic mixing in 2D V/Cr oxides, we compile the properties of the respective binary parent oxides first. Vanadium oxide on Pt(111) crystallizes in a V_2_O_3_ hc-structure in the monolayer limit but forms a vanadyl-terminated V_2_O_3_(0001) corundum lattice at higher coverage.^34^ STM images of the V_2_O_3_ monolayer that is relevant for this work exhibit large islands of irregular shape and ∼1 Å height (Figure 1a). Atomically resolved data display a hc-lattice made of interwoven V–O six rings with fcc and hcp cations having different contrasts (Figure 1a, inset). Differential conductance (d*I*/d*V*) spectra follow the typical U-shape of conductive samples with a small maximum at 0.2 V related to the V_2_O_3_ monolayer (see Supporting Information).

**Figure 1:**
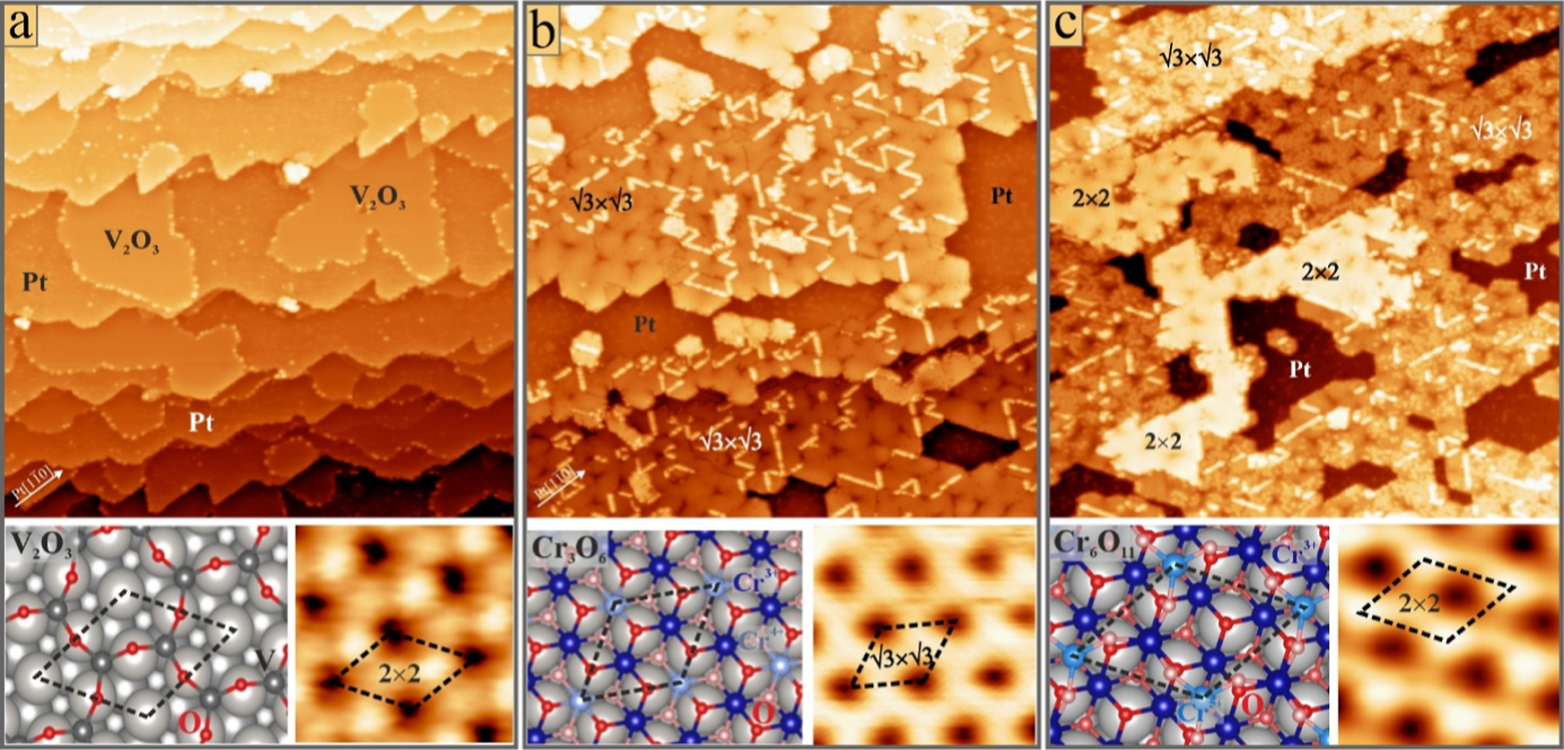
Overview (100 × 100 nm^2^, *U*_B_ = 1 V, *I* = 0.1 nA) and high-resolution STM images (1.5 × 1.5 nm^2^, 0.5 V) as well as DFT structure models of (a) the V_2_O_3_ monolayer on Pt(111), (b) the Cr_3_O_6_ phase with its unique (√3×√3)­R30° reconstruction, and (c) the (2 × 2) Cr_6_O_11_ phase on Pt(111).

Cr oxide grows in two phases on Pt(111) depending on the annealing conditions. Films treated in 1 × 10^–6^ mbar O_2_ at 650 K develop a (√3×√3)­R30° superstructure that was assigned to a dense-packed O–Cr–O trilayer (Cr_3_O_6_) with a DFT-based genetic algorithm (Figure 1b).^35^ The superstructure originates from the presence of inequivalent Cr ions in the unit cell (two Cr^3+^, one Cr^4+^) that induce distinct structural and electronic changes in their environment. The Cr^4+^ ions hereby appear with reduced contrast in positive-bias STM images (Figure 1b, inset). The (√3×√3)­R30° films are pervaded by a dislocation network that reduces misfit strain with the Pt(111) support. Films vacuum-annealed at 650 K develop a (2 × 2) superstructure, being visible in both electron diffraction and STM (Figure 1c).^35^ The (2 × 2) phase is topographically higher than Cr_3_O_6_ (4.5 versus 2.8 Å) and was assigned to a double-stack Cr_6_O_11_ film consisting of an interfacial O–Cr–O trilayer and a hc top-plane. The Cr ions in the hc-plane have alternating charge states (Cr^3+^ and Cr^5+^) and topographic heights, giving rise to the pronounced (2 × 2) pattern seen in STM (Figure 1c, inset). Both Cr–O phases have their specific d*I*/d*V* fingerprints. While the Cr_3_O_6_ trilayer shows a faint peak at 0.2 V, similar to the one seen on V_2_O_3_, Cr_6_O_11_ reveals a pronounced d*I*/d*V* maximum at 0.8 V (see Supporting Information).

### 3.2 Ternary V/Cr Mixed Oxides

Based on this binary reference, V/Cr mixing was analyzed in ternary oxide films. Two preparation schemes were explored in this study: (i) Cr deposition and oxidation in 10^–6^ mbar O_2_ followed by V deposition in 5 × 10^–7^ mbar O_2_ and 650 K vacuum annealing and (ii) V deposition/oxidation followed by Cr deposition, oxidation in 1 × 10^–6^ mbar, and vacuum annealing at 650 K. We start our discussion with pathway (i), exposing the surface to Cr first. Electron diffraction of these samples reveals a sharp (2 × 2) pattern, as found for V_2_O_3_ hc- and Cr_6_O_11_ double-stack films before.^34,35^ The corresponding STM images indeed show large V_2_O_3_ monolayer patches that homogeneously cover the Pt(111) surface. Protruding islands of a 3.5 Å apparent height (4.5 Å with respect to Pt(111)) are embedded in the V_2_O_3_ monolayer (Figure 2a,b). They adopt two nucleation scenarios, either along Pt step edges or within large Pt terraces, where they form elongated patches and triangular/rhombic islands of 5–15 nm lateral size, respectively. Atomically resolved images taken on top of the protruding islands exhibit a hexagonal hole pattern with the same orientation (along Pt ⟨11̅0⟩ ) and periodicity as the V_2_O_3_ hc-lattice (2 × 2 or 5.5 Å) (Figure 2c). The two hc-patterns exhibit a phase shift of one Pt lattice constant along one/two Pt ⟨11̅0⟩ crystallographic directions. The cations surrounding the pores in the top-layer are pairwise inequivalent and exhibit a unique bias-dependent contrast (Figure 2d and Supporting Information, Figure S2). The maximum corrugation is hereby detected at bias voltages near the Fermi level, *E*_F_. For nonideal V/Cr ratios during deposition, irregular ad-clusters appear on top of the protruding islands.

**Figure 2:**
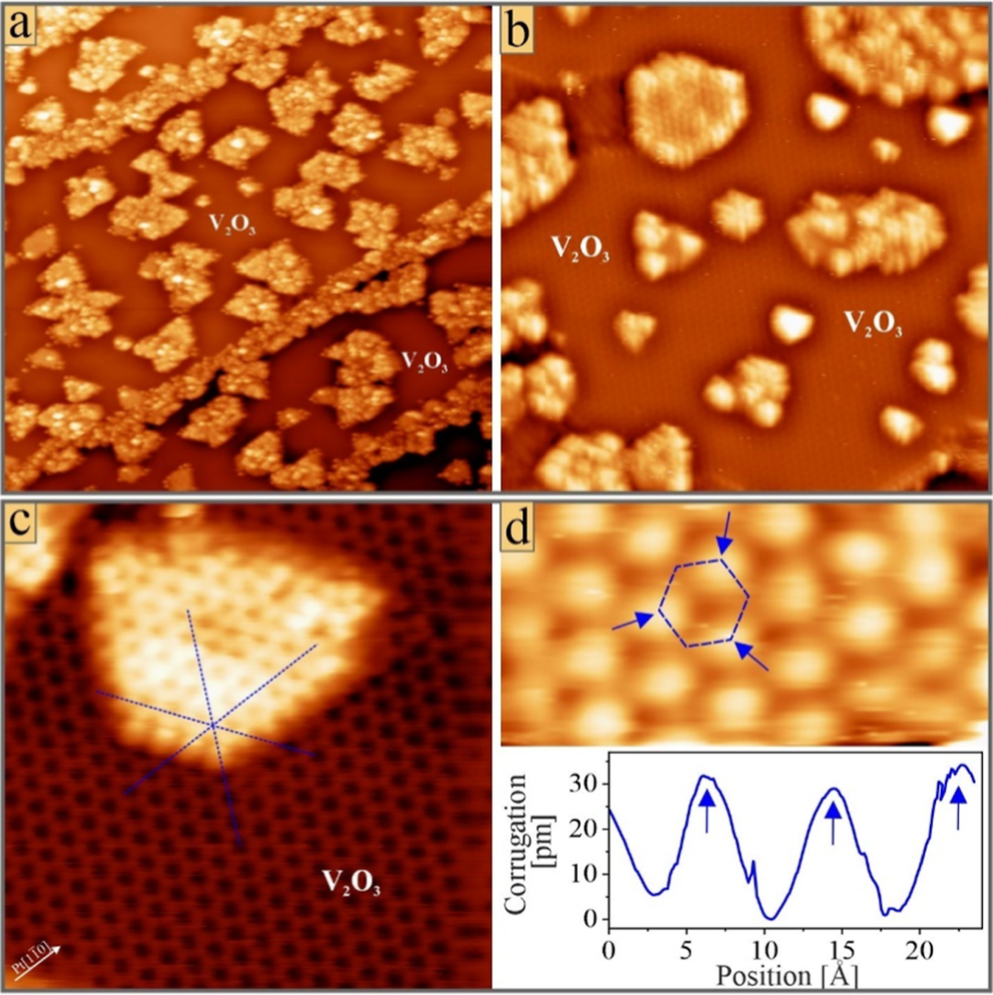
STM images of mixed oxide films on Pt(111) prepared with a Cr/V deposition sequence: (a) (100 × 100 nm^2^, *U*_B_ = 1.0 V), (b) (50 × 50 nm^2^, 0.5 V), (c) (10 × 10 nm^2^, 0.5 V), and (d) (4 × 2 nm^2^, 0.5 V) plus height profile taken along the ridge of one hc-pore. The lattice registry between the V_2_O_3_ monolayer and the mixed oxide island is illustrated with blue lines in (c).

Samples prepared by the reverse scheme, that is, V deposition first followed by Cr deposition, display a similar morphology at first glance, yet with distinct differences (Figure 3a,b). Again, 5–15 nm wide islands embedded in a homogeneous V_2_O_3_ monolayer are observed everywhere on the surface. Their apparent height of 3.5 Å above the V_2_O_3_ plane is also independent of the deposition sequence. However, only nucleation sites inside the Pt terraces are populated if V atoms are deposited first. Consequently, only triangular and rhombic islands are seen on the surface and stripe-like aggregates are absent. Further deviations emerge in the atomically resolved data of the mixed oxide islands. While islands prepared in the first scheme exhibit only a unique height level, two levels are discernible in the second scheme (Figure 3a, inset). The lower one, protruding the V_2_O_3_ monolayer by 1.75 Å, is entirely flat, and no atomic resolution is obtained. The upper level of 3.5 Å height shows a hexagonal array of pores surrounded by pairwise-different cations, as found in the first preparation method (Figure 3c,d). Our finding suggests a double-stack nature of the mixed islands. The filling of the upper level can be completed by reactive V deposition, although excess dosing leads to the formation of ad-clusters on top of the islands. As before, the hc-lattices of the V_2_O_3_ monolayer and the protruding islands exhibit a small registry shift along the Pt[11̅0] directions.

**Figure 3:**
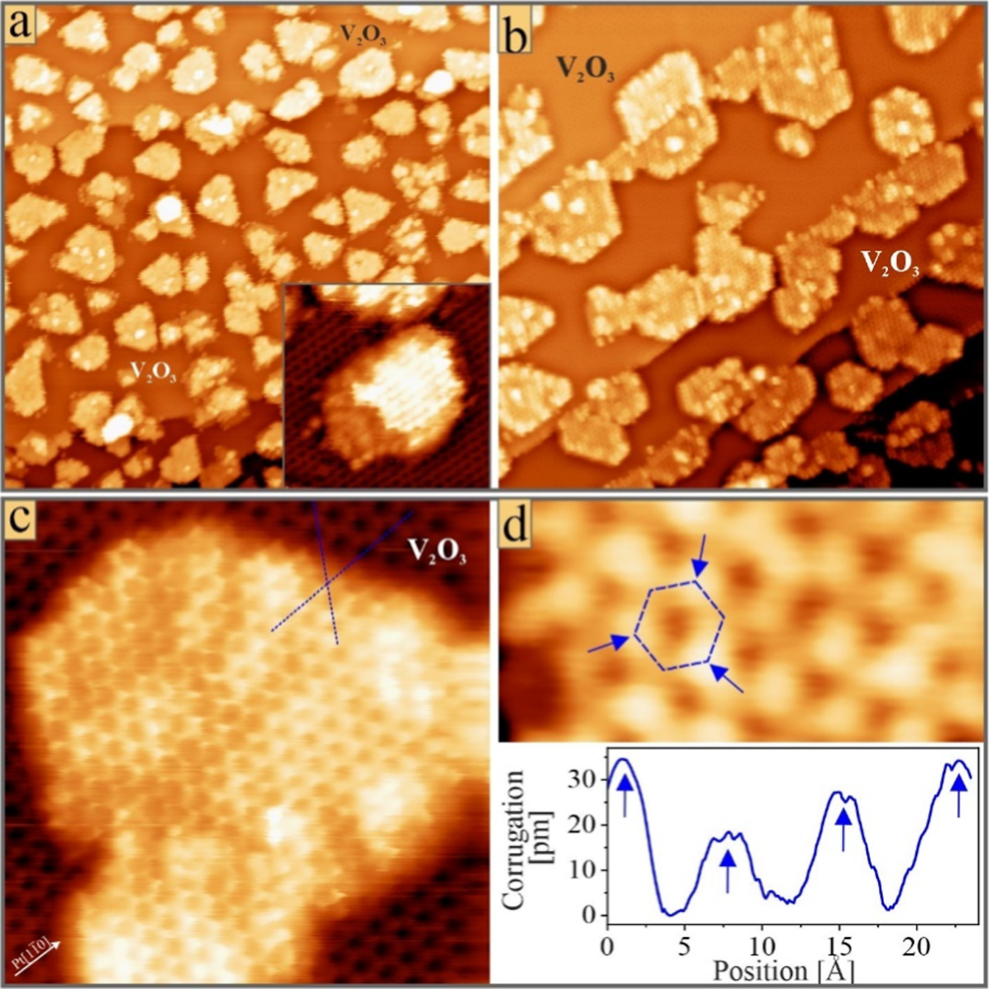
STM images of mixed oxide films prepared by a V/Cr deposition sequence: (a) (100 × 100 nm^2^, *U*_B_ = 1.5 V), (b) (50 × 50 nm^2^, 1.0 V), (c) (10 × 10 nm^2^, 0.25 V), and (d) (4 × 2 nm^2^, 0.5 V) and height profile along the ridge of the hexagonal pore marked above. The registry shift between the V_2_O_3_ monolayer and the ad-island is highlighted by the blue lines in (c). The inset in (a) shows the double-stack nature of the mixed islands (15×15 nm^2^).

## 4 Computational Results

### 4.1 Stability of Mixed Oxide Films

Following the experimental results, various structural models were explored for the V/Cr mixed oxide films on Pt(111). As a starting point, we considered configurations issued from our global optimization approach for (2 × 2) CrO_*x*_ films that comprised stacks of bilayer (Cr–O) or trilayer (O–Cr–O) structures.^35^ The bilayers were made of either dense triangular lattices of three-membered cation rings (Cr_3_O_3_) or open hc-structures of 6-membered rings (Cr_2_O_3_). The trilayers adopted dense (Cr_4_O_8_) or open Cr_3_O_6_ configurations and were located at either the interface or the surface of the film. Based on these models, we considered one, two, or three vanadium substitutions and determined their energetically favorable arrangement in the lattice. Figure 4 reports the formation energies of the most stable configurations for each composition and highlights the preferred structures as a function of the oxygen chemical potential Δμ_O_. Additional structures can be found in the Supporting Information (Figure S3).

**Figure 4:**
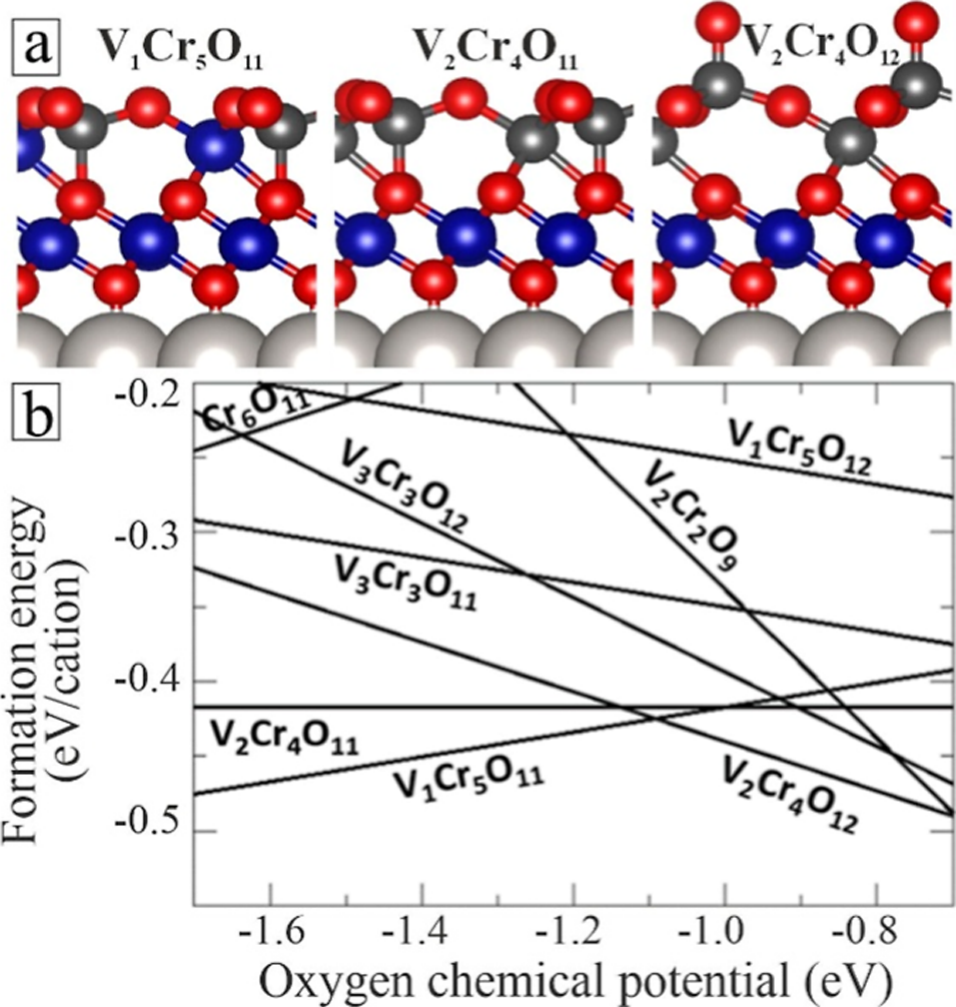
(a) Structure models and (b) formation energies as a function of the oxygen chemical potential for the most stable (2 × 2) V/Cr/O configurations on Pt(111). V, Cr, O, and Pt atoms are shown as small dark-gray, blue, red, and big gray balls, respectively.

Our analysis shows that only three configurations are thermodynamically stable in the considered range of Δμ_O_ (Figure 4). While the V_1_Cr_5_O_11_ structure is favored at O-poor conditions, the V_2_Cr_4_O_12_ is stabilized in an O-rich environment. In a small intermediate range (Δμ_O_ ∼ −1.1 eV), the V_2_Cr_4_O_11_ phase is nearly degenerate from the other two structures. All three configurations comprise with a dense O–Cr–O trilayer at the interface capped by an hc-bilayer, thus mimicking the most stable Cr_6_O_11_ and Cr_6_O_12_ binary films, yet with 50% or 100% of the surface Cr being replaced by V. In the V_1_Cr_5_O_11_ phase, the substituting V atoms occup 4-fold surface sites, and moving them to either surface or interface sites with 6-fold coordination costs 1.0 and 2.4 eV, respectively. In V_2_Cr_4_O_11_, the two V substitutes populate surface sites, while placing them at the interface exacts an energy penalty of 0.4 eV (2.3 eV) for one (both) atom. Finally, the V_2_Cr_4_O_12_ structure features a surface vanadyl bound to the 4-fold coordinated V atom, which triggers its outward relaxation and helps maintaining its local tetrahedral symmetry.

Regarding the electronic properties of the three films, the interfacial O–Cr–O trilayer contains Cr^3+^ ions in a row-wise antiferromagnetic order, while the 4-fold coordinated V ions in the surface hc-plane adopt a 5+ oxidation state (see Supporting Information, Figure S4). It is worth stressing that the presence of highly oxidized V^5+^ species is of direct interest for many oxidation catalysts, e.g., for alkane conversion.^36^ In contrast, the 6-fold coordinated ions in the hc-plane take either a 3+ (for Cr in V_1_Cr_5_O_11_), (4-δ)+ (for V in V_2_Cr_4_O_11_), or 5+ oxidation state (for V in V_2_Cr_4_O_12_). In all cases, the formal charge of the oxide double-stack amounts to −2e/cell and is compensated for by positive charging of the Pt substrate. All mixed oxide films have a negative formation energy, reflecting their thermodynamic preference over phase-separated VO_*x*_/Pt and CrO_*x*_/Pt binary films (Figure 4b). This finding aligns well with the experimental observation of mixed V/Cr oxide phases, while Cr_6_O_11_/Pt as the most stable binary phase is not detected. Note that the V_2_O_3_ binary islands only emerge due to the V excess during preparation, despite their unfavorable energetics with respect to the mixed phases.

### 4.2 STM Signature

Experimental STM images are in good agreement with Tersoff–Hamann electron density plots of the V_1_Cr_5_O_11_ and V_2_Cr_4_O_11_ models, both exposing a surface hc-plane with two inequivalent cationic sites (Figure 5a). Also, the double-stack nature of the islands with 4.5 Å topographic height matches the calculated properties of the V_*n*_Cr_6–*n*_O_11_ structures. The V=O-terminated V_2_Cr_4_O_12_ phase could not be identified in our experiments, as no protruding features corresponding to vanadyl groups could be detected in the STM.^13^ In fact, preparing V/Cr oxide films at O-rich conditions leads to a plethora of new phases, among them O-rich V_2_O_*x*_ (3 < *x* < 5)^37^ and CrO_2_ films that will be discussed in a forthcoming paper. There remains a question whether the V-poor V_1_Cr_5_O_11_ or the V-rich V_2_Cr_4_O_11_ phase gets realized at low oxygen pressures in the experiment. The two phases can be distinguished by comparing their bias-dependent corrugation, hence the height difference between 4-fold and 6-fold-coordinated sites in the hc-plane (Figures 2d and 3d), with theoretical values derived from Tersoff–Hamann plots (Figure 5b). In V_1_Cr_5_O_11_ simulations, surface V appears systematically brighter than the Cr ions in a wide bias window around *E*_F_. On V_2_Cr_4_O_11_, the height difference is small at low bias but increases at 1.5 V when the 4-fold coordinated V ions develop higher state density (see Supporting Information, Figure S5). In the STM bias series, the height difference between inequivalent cations in the hc-rings peaks at ∼0.25 V (0.4 Å) and declines with increasing positive and negative bias (Figure 5b). Despite some ambiguity due to changing STM tip states, this convincingly reproduces the calculated STM signature of the energetically preferred V_1_Cr_5_O_11_ phase.

**Figure 5:**
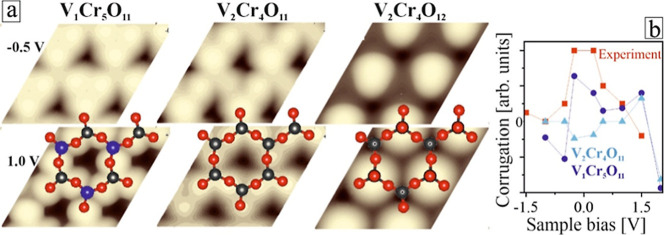
(a) Tersoff–Hamann simulations of the most stable V/Cr mixed oxide phases for two distinct bias voltages. The atomic structure models are superimposed (V: gray, Cr: blue, O: red). (b) Comparison of the measured bias-dependent corrugation within the hc-rings and simulated data for V-poor and V-rich alloy structures.

## 5 Mixing Mechanism

To elucidate the origin of the pronounced thermodynamic bias to form V/Cr mixed oxide films (Figure 4), we first recall the characteristics of the parent (√3×√3)­R30° Cr_3_O_6_/Pt film that comprises a central plane of Cr^3+^ and Cr^4+^ ions sandwiched between two oxygen planes (see Supporting Information, Section S5). The negative-charge deficit with respect to the ideal CrO_2_ stoichiometry is compensated by an electron transfer from the Pt substrate of ∼0.25 e/Cr, stabilizing the metal/oxide interface by *E*_adh_ = 0.73 eV/CrO_2_. This charge transfer combined with the O–Cr–O trilayer structure leads to an exceptionally high work function, ϕ_CrO_2_/Pt_ = 8 eV, making the CrO_2_/Pt system susceptible to be capped by an electron-donating compound. In the binary Cr_6_O_11_ film, a Cr_2_O_3_ hc–top plane serves as an effective capping with a high adhesion energy of 5.07 eV/Cr_2_O_3_. The effect can be attributed to two factors: the formation of additional Cr–O bonds and an electron transfer from the Cr_2_O_3_ bilayer to the CrO_2_/Pt support, driven by the substantial band offset between the two systems (ϕ_Cr_2_O_3__ = 5.7 eV). The latter is reflected in the 5+ charge state of one of the two hc-cations, while simultaneously, a Cr^4+^ ion in the trilayer reduces to Cr^3+^.

The stabilization gets even stronger when capping the CrO_2_/Pt trilayer with a V_2_O_3_ hc-plane (*E*_adh_ = 6.36 eV/V_2_O_3_). Two contributions are relevant for this: the stronger V–O compared to Cr–O bonds and the larger charge transfer due to an increased band offset between V_2_O_3_ (ϕ_V_2_O_3__ = 4.0 eV) and the CrO_2_/Pt support. The observed wetting of the CrO_2_/Pt trilayer by a V_2_O_3_ hc-plane clearly indicates stronger V_2_O_3_ adhesion to CrO_2_/Pt than to bare Pt (*E*_adh_ = 3.76 eV/V_2_O_3_). Regarding the composition of the capping hc-layer, previous studies revealed a moderate bias for V/Cr mixing in both freestanding and metal-supported VCrO_3_ hc-films, with mixing energies between −0.2 and −0.3 eV/VCrO_3_ relative to the Cr_2_O_3_ and V_2_O_3_ parents (see Supporting Information Section S6).^8,9^ Our calculations find this trend to be significantly enhanced on CrO_2_/Pt, a considerably more electronegative support, as seen from the much higher mixing energy of −0.87 eV/VCrO_3_. This high negative mixing energy is consistent with the thermodynamic stability of the mixed V_1_Cr_5_O_11_ configuration in a wide region of the phase diagram.

Our results also indicate a pronounced site effect, related to the coexistence of 4-fold, tetrahedral (t) and 6-fold, octahedral (o) sites in the surface hc-plane.^11^ With respect to the most stable V^*t*^Cr^*o*^O_3_/Cr_4_O_8_/Pt structure, the inverse configuration with V in octahedral and Cr in tetrahedral sites (V^*o*^Cr^*t*^O_3_/Cr_4_O_8_/Pt) is ∼1 eV higher in energy and has a positive mixing energy of +0.15 eV. Also, the electronic structure changes, as the site switch induces a redox process among the surface cations (V^5+^Cr^3+^ → V^4+^Cr^4+^), generating an unfavorable electrostatic contribution to mixing.^10^ The redox process is enabled by the overlap of nonbonding, cationic states near *E*_F_ and triggers a gap closure connected to an energy increase (Table S3, Figure S7). Interestingly, the mean cation–oxygen bond lengths are noticeably different for surface tetrahedral and octahedral sites, measuring 1.78 and 1.97 Å, respectively, regardless of the nature of the cation. Comparing these bond lengths to tabulated ionic radii,^38^ it is evident that they agree best with V^5+^ or Cr^4+^ cations in the tetrahedral and V^4+^ or Cr^3+^ in the octahedral sites.

Finally, a structural correlation can be established between the observed V_1_Cr_5_O_11_/Pt film and a spinel crystal phase. As illustrated in Figure 6, the (111) cut through an AB_2_O_4_ bulk spinel, composed of tetrahedrally (A) and octahedrally (B) coordinated cations, yields an A_2_B_4_O_12_ double-stack comprising a B_3_O_8_ trilayer capped with an A_2_BO_4_ plane. By removing the single-coordinated O atom from the latter, an inward relaxation of the adjacent A-cation into a vacant octahedral site of the trilayer takes place (see the arrows in Figure 6). This results in a double-stack made of a dense B_4_O_8_ trilayer and an ABO_3_ hc-bilayer on top, in line with the thermodynamically stable V_1_Cr_5_O_11_/Pt phase with A and B sites occupied by V and Cr, respectively. The mixed V_1_Cr_5_O_11_ film may thus be viewed as a relaxed cut through a spinel lattice, stabilized with respect to a hypothetical VCr_2_O_4_ bulk phase by excess oxygen and a charge transfer from the Pt support to enable the formation of tetrahedral V^5+^ and octahedral Cr^3+^ cations. The resulting structure might be described as V/Cr nanospinel, a unique configuration with no bulk equivalent that gets stabilized solely by its nanoscale thickness and a strong coupling to the Pt(111) support.

**Figure 6:**
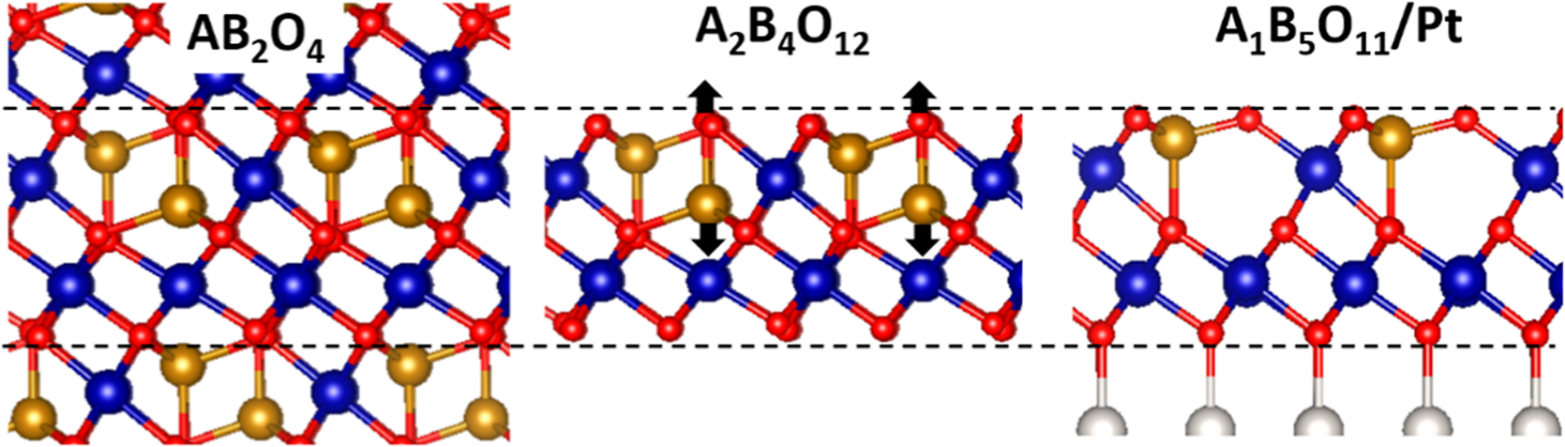
Structural link between an AB_2_O_4_ bulk spinel (left) and the observed V_1_Cr_5_O_11_/Pt film (right). Four- (A) and 6-fold coordinated (B) cations are plotted as yellow and blue balls, whereas oxygen and Pt atoms are shown with red and gray colors, respectively. Removal of the single coordinated oxygen atom and inward relaxation of the corresponding A cation (see arrows) lead to the film structure observed in the experiment.

To gain insight into the stabilization of other nanospinels, we have investigated two analogous mixed bistacks: VMn_5_O_11_/Pt and TiCr_5_O_11_/Pt (Supporting Information, Table S3). The former exhibits an internal charge distribution and favorable substitution energy similar to those of VCr_5_O_11_/Pt. This indicates that vanadium is effectively stabilized also in Mn-based spinel films and might be incorporated even into a broader class of TM-based ultrathin films. In contrast, the TiCr_5_O_11_/Pt system shows a less favorable mixing energy, as the surface Ti ions adopt a 4+ charge state, while higher oxidation states are inaccessible. This underscores the critical role of charging effects to stabilize the nanospinel configuration.

## 6 Conclusions

Low-temperature STM measurements demonstrated the mixing of V and Cr ions into a double-stack oxide film of 4.5 Å thickness, terminated by a surface hc-layer. Associated DFT calculations identified the most favorable film structure to comprise a densely packed O–Cr–O trilayer at the interface and a VCrO_3_ hc-plane at the surface. The resulting configuration shows an excellent match with the STM data. Moreover, it is thermodynamically stable across a wide range of oxygen chemical potentials relevant for the experiment. Our calculations reveal two driving forces for V/Cr mixing in the surface plane: a sizable electron transfer toward the interface trilayer and the presence of two distinct surface sites being occupied by tetrahedrally coordinated V^5+^ and octahedrally coordinated Cr^3+^ cations. The mixed oxide film can be viewed as a 2D cut through a hypothetical V/Cr spinel, a unique ultrathin oxide phase that emerges due to its strong interaction with the Pt(111) below.

## Supplementary Material



## Notes

STM conductance spectroscopy of V/Cr mixed oxide films and their binary parent oxides, bias-dependent STM images of V/Cr mixed oxide films and their binary parent oxides, compilation of all structural models considered in this work, electronic properties of V/Cr mixed oxide films from DFT, DFT characteristics of the CrO_2_/Pt and VCrO_3_ honeycomb bilayers, site effects relevant for V/Cr mixing in the hc plane of V_1_Cr_5_O_11_ on Pt(111), and stability of other nanospinel structures, such as VMn_5_O_11_/Pt and TiCr_5_O_11_/Pt (PDF)

## References

[1] Li Y. Y., Zhang Y. S., Qian K., Huang W. X. (2022). Metal-Support Interactions
in Metal/Oxide Catalysts and Oxide-Metal Interactions in Oxide/Metal
Inverse Catalysts. ACS Catal..

[2] Luo Z. X., Zhao G. Q., Pan H. G., Sun W. P. (2022). Strong
Metal-Support
Interaction in Heterogeneous Catalysts. Adv.
Energy Mater..

[3] Stavale F., Shao X., Nilius N., Freund H.-J., Prada S., Giordano L., Pacchioni G. (2012). Donor Characteristics
of Transition-Metal-Doped
Oxides: Cr-Doped MgO versus Mo-Doped CaO. J.
Am. Chem. Soc..

[4] Włodarczyk R., Sauer J., Yu X., Boscoboinik J. A., Yang B., Shaikhutdinov S., Freund H. J. (2013). Atomic Structure
of an Ultrathin Fe-Silicate Film Grown on a Metal: A Monolayer of
Clay?. J. Am. Chem. Soc..

[5] Curto A., Sun Z., Rodriguez-Fernández J., Zhang L., Parikh A., Tan T., Lauritsen J. V., Vojvodic A. (2019). Anisotropic Iron-Doping Patterns
in Two-Dimensional Cobalt Oxide Nanoislands on Au(111). Nano Res..

[6] Schenk S., Krahn O., Cockayne E., Meyerheim H. L., de Boissieu M., Förster S., Widdra W. (2022). 2D Honeycomb Transformation
into Dodecagonal Quasicrystals Driven by Electrostatic Forces. Nat. Commun..

[7] Pomp S., Kuhness D., Barcaro G., Sementa L., Mankad V., Fortunelli A., Sterrer M., Netzer F. P., Surnev S. (2016). Two-Dimensional
Iron Tungstate: A Ternary Oxide Layer with Honeycomb Geometry. J. Phys. Chem. C.

[8] Goniakowski J., Noguera C. (2020). Properties of Metal-Supported Oxide Honeycomb Monolayers:
M_2_O_3_ and MM′O_3_ on Me (111)
(M, M′ = Ti, V, Cr, Fe; Me = Ag, Au, Pt). J. Phys. Chem. C.

[9] Goniakowski J., Noguera C. (2019). Intrinsic Properties
of Pure and Mixed Monolayer Oxides
in the Honeycomb Structure: M_2_O_3_ and MM′O_3_ (M, M′ = Ti, V, Cr, Fe). J.
Phys. Chem. C.

[10] Wemhoff P. I., Nilius N., Noguera C., Goniakowski J. (2022). Two-Dimensional
Oxide Alloys Probed at the Atomic Level: (V,Fe)_2_O_3_ Honeycomb Monolayers on Pt(111). J. Phys.
Chem. C.

[11] Wemhoff P. I., Nilius N., Noguera C., Goniakowski J. (2022). Structure
and Stoichiometry Self-Organization in a Mixed Vanadium–Iron
Oxide Honeycomb Film on Ru(0001). J. Phys. Chem.
C.

[12] Goniakowski J., Wemhoff P. I., Nilius N., Noguera C. (2023). Stability and Mixing
Behavior of Vanadium-Iron Oxide Monolayers on Pt(111) and Ru(0001). J. Phys.: Condens. Matter.

[13] Netzer, F. P. ; Noguera, C. Oxide Thin Films and Nanostructures; Oxford University Press, 2021.

[14] Muthuselvam I. P., Bhowmik R. N. (2009). Structural
Phase Stability and Magnetism in Co_2_FeO_4_ Spinel
Oxide. Solid
State Sci..

[15] Cai Q., Wang J. G., Wang Y., Mei D. (2016). First-Principles Thermodynamics
Study of Spinel MgAl_2_O_4_ Surface Stability. J. Phys. Chem. C.

[16] Zhang L., Kuhn M., Diebold U. (1997). Growth, Structure and Thermal Properties
of Chromium Oxide Films on Pt(111). Surf. Sci..

[17] Priyantha W. A. A., Waddill G. D. (2005). Structure of Chromium
Oxide Ultrathin Films on Ag(111). Surf. Sci..

[18] Maetaki A., Yamamoto M., Matsumoto H., Kishi K. (2000). The Preparation of
Ultra-Thin Chromium-Vanadium Oxides on Cu(100) Studied by XPS and
LEED. Surf. Sci..

[19] Mohammadi M., Negreiros F. R., Radlinger T., Edelmayer P., Netzer F. P., Surnev S. (2022). Interaction of Na with
2D WO_3_ and MoO_3_ Layers on Pd(100): From Doping
to 2D
Bronze Formation. J. Phys. Chem. C.

[20] Kresse G., Hafner J. (1993). Ab-initio Molecular Dynamics for
Liquid Metals. Phys. Rev. B.

[21] Kresse G., Furthmuller J. (1996). Efficient Iterative Schemes for ab-initio Total Energy
Calculations with a Plane-Wave Basis Set. Phys.
Rev. B.

[22] Blöchl P. E. (1994). Projector
Augmented-Wave Method. Phys. Rev. B.

[23] Kresse G., Joubert D. (1999). From Ultrasoft Pseudopotentials
to the Projector Augmented-Wave
Method. Phys. Rev. B.

[24] Dion M., Rydberg H., Schroder E., Langreth D. C., Lundqvist B. I. (2004). Van der
Waals Density Functional for General Geometries. Phys. Rev. Lett..

[25] Klimes J., Bowler D. R., Michaelides A. (2011). Van der Waals
Density Functionals
Applied to Solids. Phys. Rev. B.

[26] Anisimov V. I., Aryasetiawan F., Lichtenstein A. I. (1997). First Principles
Calculations of
the Electronic Structure and Spectra of Strongly Correlated Systems:
the LDA+U Method. J. Phys.: Condens. Matter.

[27] Dudarev S. L., Botton G. A., Savrasov S. Y., Humphreys C. J., Sutton A. P. (1998). Electron-Energy-Loss Spectra and
the Structural Stability
of Nickel Oxide: An LSDA+U Study. Phys. Rev.
B.

[28] Le H.-L. T., Goniakowski J., Noguera C. (2018). Properties of Mixed Transition Metal
Oxides: MM’O_3_ in Corundum-type Structures (M, M’
= Al, Ti, V, Cr, and Fe). Phys. Rev. Mater..

[29] Goniakowski J., Noguera C. (2019). Properties of M_2_O_3_/Au­(111) Honeycomb
Monolayers (M = Sc, Ti, V, Cr, Mn, Fe, Co, Ni). J. Phys. Chem. C.

[30] Bader R. F. W. (1991). A Quantum
Theory of Molecular Structure and its Applications. Chem. Rev..

[31] Henkelman G., Arnaldsson A., Jonsson H. (2006). A Fast and Robust Algorithm for Bader
Decomposition of Charge Density. Comput. Mater.
Sci..

[32] Tersoff J., Hamann D. R. (1985). Theory of the Scanning
Tunneling Microscope. Phys. Rev. B.

[33] Momma K., Izumi F. (2011). VESTA 3 for Three-dimensional Visualization of Crystal, Volumetric
and Morphology Data. J. Appl. Crystallogr..

[34] Surnev S., Vitali L., Ramsey M., Netzer F., Kresse G., Hafner J. (2000). Growth and Structure
of Ultrathin Vanadium Oxide Layers
on Pd(111). Phys. Rev. B.

[35] Missaoui G., Wemhoff P. I., Noguera C., Goniakowski J., Nilius N. (2024). Chromium Oxide Thin Films on Pt(111): An STM and DFT
Excursion through the Phase Diagram. J. Phys.
Chem. C.

[36] Brücher O., Hartung J. (2011). Vanadium­(V)-Catalyzed Oxidative Bromination of Acid
Labile Alkenols and Alkenes in Alkyl Carbonates. ACS Catal..

[37] Missaoui G., Wemhoff P. I., Nilius N. (2024). Atomic Scale
Insights into Reversible
Oxygen Storage in Vanadium Oxide Thin Films. ChemPhysChem.

[38] Shannon R. D. (1976). Revised
Effective Ionic Radii and Systematic Studies of Interatomic Distances
in Halides and Chalcogenides. Acta Crystallogr..

