# Supplementary material for: Chromium/Vanadium Mixed Oxide Films on Pt(111): Revealing Oxide Alloying Mechanisms in Two Dimensions
Source: ACS Appl Mater Interfaces. 2025 Jul 25;17(31):45026–32. doi: 10.1021/acsami.5c05932 (PMC12367229; doi:10.1021/acsami.5c05932)
Supplement: Supplementary file 1 [file am5c05932_si_001.pdf]

Supporting Information

Chromium/Vanadium Mixed Oxide Films on Pt(111): Revealing Oxide Alloying Mechanisms in Two Dimensions

Ghada Missaoui,[a] Piotr Igor Wemhoff,[a] Jacek Goniakowski,[b] Claudine Noguera,[b] and Niklas Nilius [a,]*

[a] Carl von Ossietzky Universität Oldenburg, Institut für Physik, D-26111 Oldenburg, Germany
[b] CNRS-Sorbonne Université, Institut des Nanosciences de Paris, UMR 7588, F-75005 Paris, France

Corresponding authors: jacek.goniakowski@insp.jussieu.fr, niklas.nilius@uol.de

S1. Conductance signature of ternary V/Cr mixed oxide films

STM conductance spectroscopy has been employed to probe the local density of states (DOS) of the mixed oxide films around E_F. The V_2O_3 and Cr_3O_6 binary oxide films show rather featureless dI/dV spectra, characterized by an overall *U*-shape and a shallow maximum at 0.2 V (Fig. S1).[1,2] The Cr_6O_{11} data, on the other hand, exhibit a pronounced dI/dV maximum at 0.8 V, being assigned to the 3d states of Cr^{5+} ions in the top hc-plane.[2] Conductance spectra of the protruding V/Cr mixed oxide islands appear as convolution of the two spectral types. They exhibit the *U*-shape of the binary films in addition to a weak 0.8 V shoulder. Evidently, the Cr^{5+} ions are largely replaced by surface V ions, following the proposed $V_1Cr_5O_{11}$ mixed oxide structure.

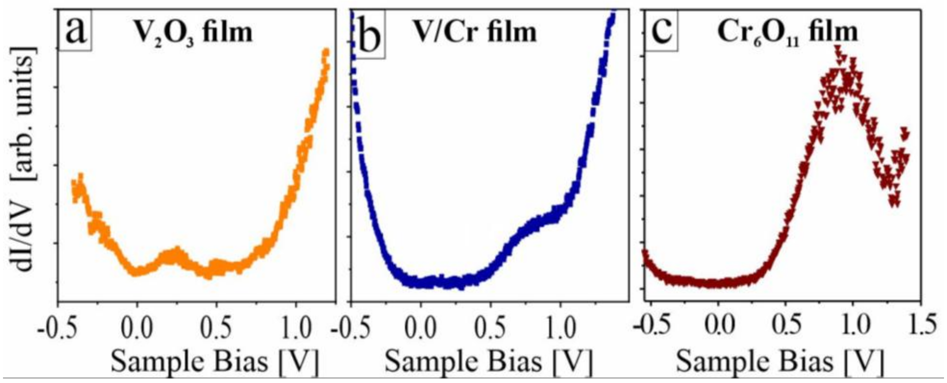

Fig. S1: STM conductance spectra of **(a)** the V_2O_3 honeycomb monolayer, **(b)** the V/Cr mixed oxide islands and **(c)** the Cr_6O_{11} double-stack film. The bias setpoint was set to 1.5 V in all spectra.

S2. Distinction between binary Cr_6O_{11} and ternary $V_1Cr_5O_{11}$ films

Experimental data and DFT calculations convincingly indicate that V/Cr alloying on Pt(111) preferentially takes place on the basis of the Cr_6O_{11} double-stack structure, comprising an interfacial O-Cr-O tri-layer and a surface honeycomb (hc) plane. Upon alloying, the fourfold-coordinated Cr ions in the hc-plane get replaced by V, resulting in a $V_1Cr_5O_{11}$ structure, while formation of a V-rich $V_2Cr_4O_{11}$ phase seems incompatible with the experimental data. To corroborate this conclusion, the bias-dependent STM signature of V/Cr mixed oxide films needs to be distinguished from the one of binary Cr_6O_{11}, which is the purpose of this paragraph.

Figure S2a displays a bias series taken on an alloy film prepared by sequential Cr/V deposition. A simple hexagonal pattern appears throughout the series, yet with a clear contrast difference between the in-equivalent sites of the top hc-plane. As discussed in the main text, this cationic corrugation peaks at E_F

and declines when moving to higher positive and negative bias. Figure S2b, showing a similar bias series but taken on binary Cr_6O_{11}, reveals a different STM contrast behavior than the alloy film.[2] While the Kagome structure of the O sublattice dominates at negative bias, a simple hc-film appears at voltages close to E_F. With increasing positive bias, a distinct (2×2) contrast becomes visible, produced by Cr^{5+} ions in the top hc-plane that exhibit higher empty DOS hence larger topographic height than their Cr^{3+} counterparts. The resulting bias dependence of the cationic corrugation, also plotted in Fig. S2c, is distinctively different from the one of the alloy films. These distinct bias signatures thus render binary Cr_6O_{11} and ternary $V_1Cr_5O_{11}$ films experimentally distinguishable, further strengthening our assignment of the V/Cr mixed-oxide phase.

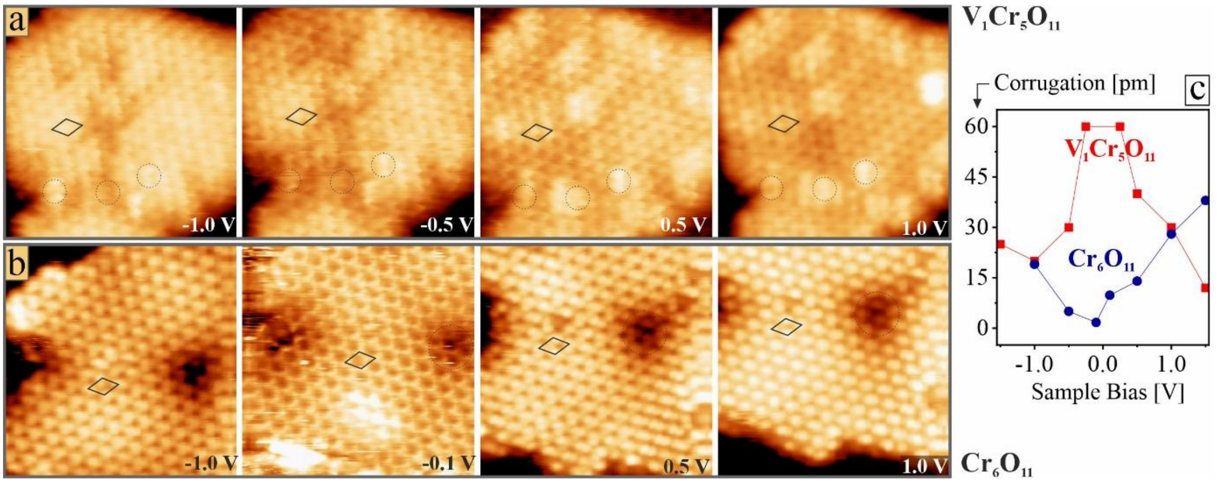

Fig. S2: Bias-dependent STM images of **(a)** protruding islands in V/Cr mixed oxide films and **(b)** a (2×2) Cr_6O_{11} domain, both prepared on Pt(111) (8×8 nm^2, I = 0.25 nA). The unit cell as well as distinct image features are marked by solid and dashed symbols. **(c)** Extracted bias-dependent corrugation of hc-rings on both surfaces, showing a distinctively different behavior.

S3. Considered structure models

Global optimization of pure (2×2) CrO_x/Pt(111) films revealed a building scheme involving double-stacks of bilayer (Cr−O) or trilayer (O−Cr−O) structures.[2] As schematized in Fig. S3, the bilayers take the form of either dense triangular lattices with 3-membered cation rings (Cr_3O_3 and Cr_4O_4), or open honeycomb lattices, characterized by 6-membered rings (Cr_2O_3). The trilayers adopt either more (Cr_4O_8) or less dense (Cr_3O_8) configurations derived from Cr_4O_4 bilayers, or a much more open structure (Cr_2O_6) based on a hc-bilayer Cr_2O_3.

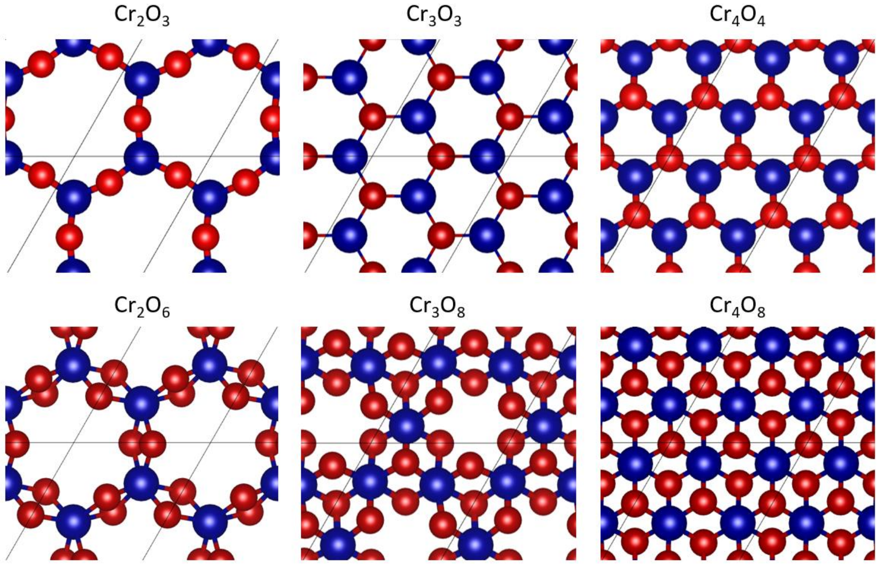

Figure S3: Atomic structures of different bi- and trilayers that are compatible with (2×2) CrO_x/Pt(111) double-stacks (top view). Top panels: Cr_2O_3, Cr_3O_3, and Cr_4O_4 bilayers; bottom panels: Cr_2O_6, Cr_3O_8, and Cr_4O_8 trilayers. Anions and cations are shown with red and blue balls, respectively, and (2×2) cells are indicated.

The thermodynamically most stable Cr_6O_{11} double-stack comprises a dense Cr_4O_8 interface trilayer capped with a Cr_2O_3 hc-bilayer. At oxygen-rich conditions, a surface chromyl group may form in addition, resulting in a Cr_6O_{12} configuration. Global optimization revealed other low-energy structures, including a Cr_6O_{12} configuration with a less dense Cr_3O_8 interface trilayer and a dense Cr_3O_3 surface bilayer, featuring again a surface chromyl group (Cr_3O_4). Moreover, a Cr_6O_9 configuration composed of an interface Cr_3O_6 trilayer and a Cr_3O_3 surface bilayer was identified. Lastly, Cr_4O_9 and Cr_5O_9 configurations arise from combining a Cr_2O_6 interfacial trilayer with either a Cr_2O_3 hc-bilayer or a Cr_3O_3 dense bilayer, respectively.[2]

Based on these configurations, we considered one, two, or three vanadium substitutions and determined their energetically most favorable arrangement within the film. Complementing the thermodynamically stable configurations presented in Fig.4 of the main text, Fig. S4 displays the atomic structures of additional low-energy configurations. They include a $V_3Cr_3O_{12}$ film comprising a $VCr_2O_4/V_2CrO_8/Pt$ double-stack, as well as $V_3Cr_3O_9$ ($V_3O_3/Cr_3O_6/Pt$), $V_2Cr_2O_9$ ($V_2O_3/Cr_2O_6/Pt$), and $V_2Cr_3O_9$ ($VCr_2O_3/VCrO_6/Pt$) configurations.

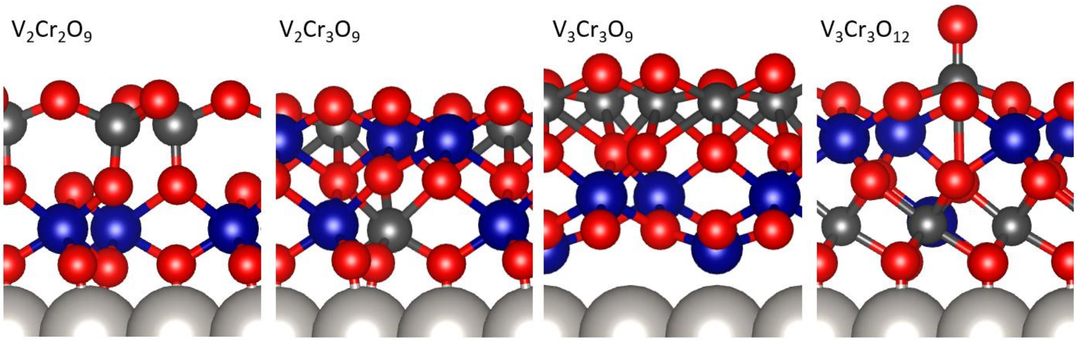

Figure S4: Side views of selected low-energy configurations of different stoichiometry and vanadium content: $V_2Cr_2O_9$, $V_2Cr_3O_9$, $V_3Cr_3O_9$, and $V_3Cr_3O_{12}$. V, Cr, O and Pt atoms are shown as small dark-grey, blue, red, and big grey balls, respectively.

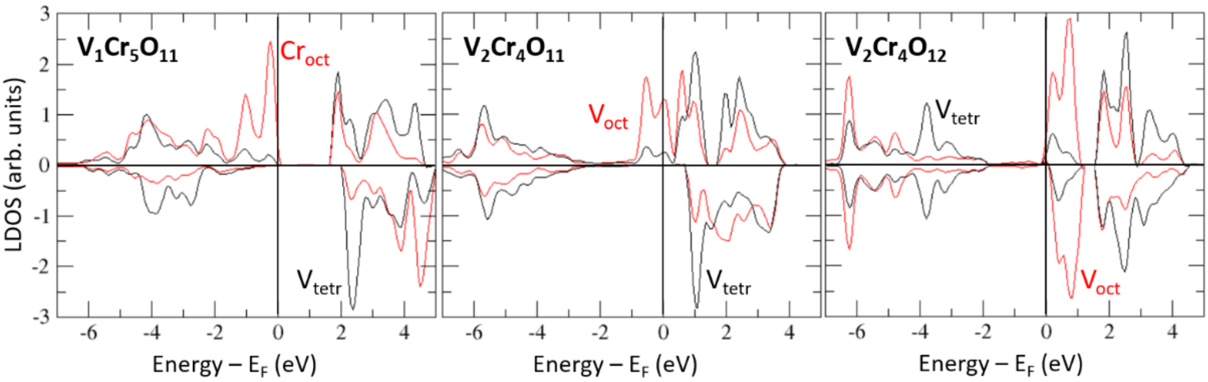

Figure S5: Local density of states (LDOS) of cations in tetrahedral (black lines) and octahedral (red lines) environments in the capping hc-bilayer in the thermodynamically stable configurations: $V_1Cr_5O_{11}/Pt$, $V_2Cr_4O_{11}/Pt$, and $V_2Cr_4O_{12}/Pt$.

S4. Electronic characteristics of the thermodynamically stable mixed films

The three thermodynamically most stable alloy configurations (Fig. 4 in the main text) display distinct electronic properties, although the dense O-Cr-O trilayer at the interface involves row-wise antiferromagnetically ordered Cr cations in 3+ oxidation state in all cases (q_{Cr} ~ +1.7 e, μ_{Cr} ~ ±2.7 μ_B). In the capping hc-bilayer, the 4-fold coordinated V cations systematically adopt a 5+ oxidation state (q_V ~ +2.1 e, μ_V ~ 0.0 μ_B), whereas the 6-fold coordinated surface cation is either Cr^{3+} (q_{Cr} ~ +1.8 e, μ_{Cr} ~ 2.8 μ_B) in

the $V_1Cr_5O_{11}$ phase, $V^{(4-\delta)+}$ (q_V ~ +2.0 e, μ_V ~ 1.4 μ_B) in the $V_2Cr_4O_{11}$ phase, or V^{5+} (q_V ~ +2.1 e, μ_V ~0.0 μ_B) in the $V_2Cr_4O_{12}$ configuration. This confers a pronounced insulating character to the capping $VCrO_3$ and V_2O_4 hc-bilayers in the $V_1Cr_5O_{11}$ and $V_2Cr_4O_{12}$ configurations, respectively, with a gap of nearly 2 eV, while the V_2O_3 plane in the $V_2Cr_4O_{11}$ configuration is clearly metallic (Fig. S5). In all cases, the formal charge of the oxide double-stacks amounts to -2e/cell and is compensated by a positive charging of the Pt support (q_{Pt} ~ 0.8–0.9 e/cell). It is worth stressing that the results of hybrid calculations, presented in the bottom panel of Tab. S1, fully confirm the electronic characteristics extracted from GGA+U results.

**GGA+U**	q_V/q_{Cr} (e)	μ_V/μ_{Cr} (μ_B)	q_{Pt} (e/cell)	Q_V/Q_{Cr}
$V_1Cr_5O_{11}$/Pt	2.12, 1.83<br>1.69,1.72,1.69,1.69	0.1, 2.8<br>-2.7, -2.7, 2.7, 2.7	+0.90	$V^{5+}Cr^{3+}$<br>$Cr^{3+}Cr^{3+}Cr^{3+}Cr^{3+}$
$V_2Cr_4O_{11}$/Pt	2.11, 2.01<br>1.68,1.71,1.68,1.68	0.3, 1.4<br>-2.7, -2.7, 2.7, 2.7	+0.81	$V^{(5-\delta)+}V^{(4-\delta)+}$<br>$Cr^{3+}Cr^{3+}Cr^{3+}Cr^{3+}$
$V_2Cr_4O_{12}$/Pt	2.25, 2.13<br>1.68,1.72,1.69,1.69	0.1, 0.0<br>-2.7, -2.7, 2.7, 2.7	+0.92	$V^{5+}V^{5+}$<br>$Cr^{3+}Cr^{3+}Cr^{3+}Cr^{3+}$

**hybrid**	q_V/q_{Cr} (e)	μ_V/μ_{Cr} (μ_B)	q_{Pt} (e/cell)	Q_V/Q_{Cr}
$V_1Cr_5O_{11}$/Pt	2.24, 1.93<br>1.78,1.82,1.79,1.79	0.1, 2.8<br>-2.7, -2.7, 2.7, 2.7	+0.98	$V^{5+}Cr^{3+}$<br>$Cr^{3+}Cr^{3+}Cr^{3+}Cr^{3+}$
$V_2Cr_4O_{11}$/Pt	2.25, 2.09<br>1.78,1.81,1.78,1.78	0.2, 1.6<br>-2.7, -2.7, 2.7, 2.7	+0.89	$V^{5+}V^{(4-\delta)+}$<br>$Cr^{3+}Cr^{3+}Cr^{3+}Cr^{3+}$
$V_2Cr_4O_{12}$/Pt	2.40, 2.26<br>1.78,1.82,1.78,1.78	0.0, 0.0<br>-2.7, -2.7, 2.7, 2.7	+0.97	$V^{5+}V^{5+}$<br>$Cr^{3+}Cr^{3+}Cr^{3+}Cr^{3+}$

Table S1: Electronic properties of the three thermodynamically stable configurations obtained with GGA+U (top) and hybrid (bottom) exchange-correlation functionals: Bader cation charges q_V/q_{Cr} (e) and magnetic moments μ_V/μ_{Cr} (μ_B), Pt Bader charge q_{Pt} (e/cell) and approximate formal oxidation states of cations. For each configuration, results for surface and interface cations in the double-stacks are given in top and bottom lines, respectively.

S5. Characteristics of the CrO_2/Pt system

The thermodynamically stable O-rich (√3×√3) R30° Cr_3O_6/Pt trilayer consists of a dense Cr cation plane (involving cations in 3+ and 4+ oxidation states) sandwiched between two oxygen planes.[2] Substantial electron transfer from the Pt substrate (q_{Pt} ~ +0.037 eÅ^{-2}) compensates for the negative-charge deficit and strongly stabilizes the film (E_{adh} = 1.8 Jm^{-2}). Trilayer O-Cr-O structure and the strong interfacial charge transfer result in an exceptionally high work function ϕ = 8 eV of the CrO_2/Pt system, which constitutes a significant $\Delta\phi$ ~ +1.9 eV increase compared to bare Pt(111). These properties contrast sharply with those of the somewhat less O-rich (2×2) Cr_2O_3/Pt honeycomb bilayer, which consists of a sparse cation plane (cations essentially in 3+ oxidation state) capped by an oxygen plane. [2] Here, the Pt substrate takes a smaller and negative charge (q_{Pt} ~ -0.022 eÅ^{-2}) which produces weaker interface interactions (E_{adh} = 1.1 Jm^{-2}). Opposing charge transfer and structural (rumpling) contributions to the interface dipole only lead to a moderate modification of the Pt work function ($\Delta\phi$ ~ +0.5 eV), unlike its drastic increase in the CrO_2/Pt system.

This stark contrast stems from the distinct properties of freestanding CrO_2 and Cr_2O_3 films, particularly their electronegativity differences, which lead to dissimilar band alignments with the Pt(111) substrate (Fig. S6). Freestanding Cr_2O_3 exhibits a work function (ϕ = +5.7 eV) similar to Pt(111), resulting in minimal band offset and weak electron exchange at the interface. Conversely, the substantially higher work function of freestanding CrO_2 trilayer (ϕ = +8.1 eV) creates a 2 eV offset, prompting significant electron transfer from the metal substrate to the oxide film. These electrons fill empty states above E_F

of the CrO_2 (Fig. 6), reducing most cations from 4+ to 3+ when Pt-supported. Such exceptionally high work function of the freestanding CrO_2 film primarily arises from its trilayer structure, with positive metal ions sandwiched between negatively charged oxygen planes.

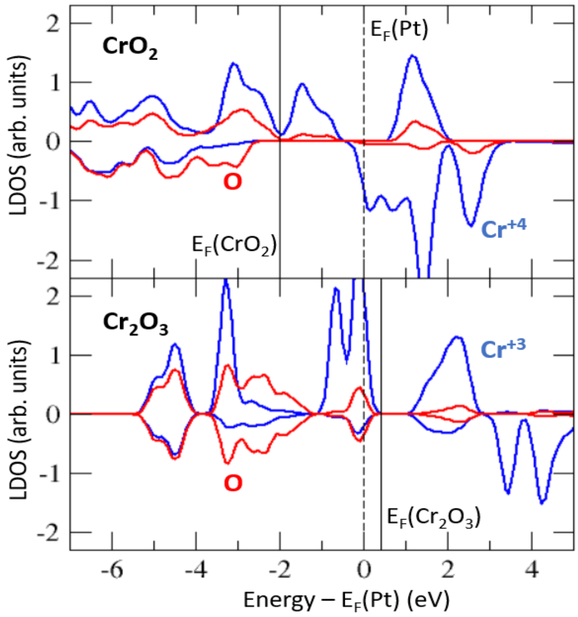

Figure S6: LDOS of anions (red) and cations (blue) in freestanding CrO_2 and Cr_2O_3 films on an energy scale obtained by aligning the common vacuum levels. Fermi-level positions of film and Pt(111) are marked by solid and dashed lines, respectively.

Finally, nanoscale film thickness and interaction with the Pt support significantly influence the relative stability of the CrO_2 and Cr_2O_3 phases. While calculations show that bulk CrO_2 (rutile) is thermodynamically favored over bulk Cr_2O_3 (corundum) only in significantly O-rich environments, $\Delta\mu_O > -0.6$ eV, its stability range tends to extend to more O-poor conditions in freestanding ($\Delta\mu_O > -3.7$ eV) and Pt-supported ($\Delta\mu_O > -3.3$ eV) trilayers. This extended stability range corresponds to the experimentally observed O-rich stoichiometries of ultrathin CrO_x films under UHV conditions, highlighting the impact of dimensionality and substrate interactions on phase stability.

S6. Mixing characteristics of $VCrO_3$ honeycomb bilayers

Previous studies predicted only a moderate bias for V/Cr mixing in freestanding and metal-supported $VCrO_3$ honeycomb bilayers, with mixing energies ranging from -0.1 to -0.3 eV/$VCrO_3$ relative to the respective freestanding or metal-supported Cr_2O_3 and V_2O_3 bilayer references.[3,4] The mixing energy is significantly enhanced in the CrO_2/Pt system, E_{mix} = -0.87 eV/$VCrO_3$, which is again the consequence of the large electronegativity ϕ_{sup} of this particular support.

	ϕ_{sup} (eV)	q_{sup} (e/fu)	Q_V/Q_{Cr}	E_{adh} (eV/fu)	E_{mix} (eV/fu)
$VCrO_3$ ([4])	-	-	$V^{4-\delta}Cr^{2+\delta}$	-	-0.14
$VCrO_3$/Pt ([3])	6.1	-0.88	$V^{5+}Cr^{3+}$	2.92	-0.27
$VCrO_3/Cr_4O_8$/Pt	8.0	-1.23	$V^{5+}Cr^{3+}$	6.43	-0.87

Table S2: Properties of freestanding, Pt- and CrO_2/Pt-supported mixed $VCrO_3$ hc-bilayers: support work function ϕ_{sup} (eV) and Bader charge q_{sup} and formal charges of cations Q_V/Q_{Cr} (e/cell), bilayer adhesion E_{adh} (eV/cell) and mixing energies E_{mix} (eV/cell).

S7. Site effects in $VCrO_3$ honeycomb bilayers

The hc-plane that caps the O-Cr-O/Pt(111) trilayer exhibits two specific binding options for cations, one with tetrahedral and one with octahedral coordination. In the energetically preferred atom arrangement,

the former one is occupied by V ions in 5+ charge state while the latter is populated by Cr^{3+} ions. Swapping this configuration, i.e. placing the V in octahedral and the Cr in tetrahedral sites increases the total energy by ~1 eV. Figure S7 displays the associated changes in the LDOS of the system, emphasizing in particular the closing of the band-gap at E_{Fermi} in the $V^{O}Cr^{t}O_3/Cr_4O_8$ configuration.

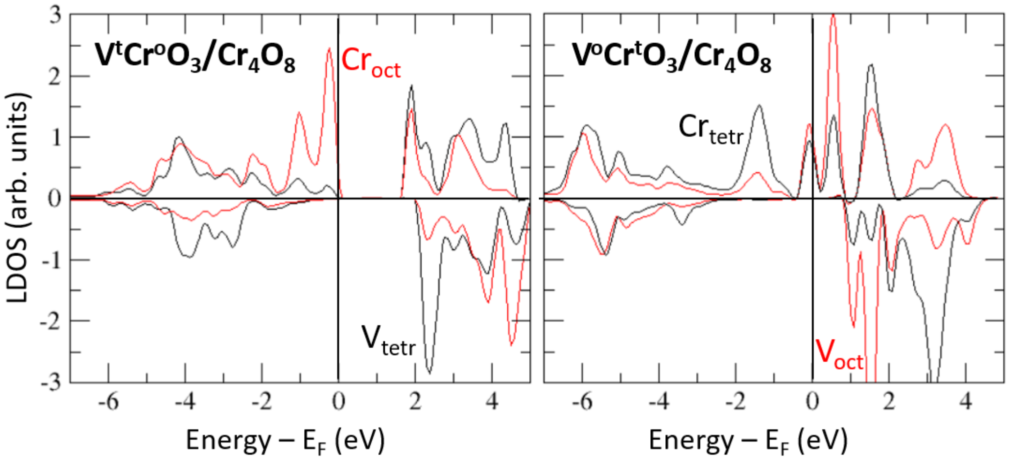

Figure S7: LDOS of the V and Cr cations in the capping hc-bilayer of the thermodynamically stable configurations $V^{t}Cr^{o}O_3/Cr_4O_8$/Pt (left) and of the inverse configuration $V^{o}Cr^{t}O_3/Cr_4O_8$/Pt in which they have exchanged sites (right). The Fermi level imposed by the Pt substrate is marked by a solid black line.

S8. Spinel-type bi-stacks with alternative chemical composition

To gain more general insights into the properties of nano-spinels, we have explored two analogous mixed bi-stacks: VMn_5O_{11}/Pt and $TiCr_5O_{11}$/Pt (Tab. S3). The VMn_5O_{11}/Pt(111) system exhibits a charge distribution highly similar to that of VCr_5O_{11}/Pt, indicating that vanadium can be easily incorporated into such Mn-based nano-spinels. In contrast, the $TiCr_5O_{11}$/Pt system features surface Ti^{4+} cations, making the stabilization of a spinel-type mixed phase unfavorable.

	E_{sub} (eV/A)	q_A/q_B (e)	μ_A/μ_B (μ_B)	q_{Pt} (e/cell)	Q_A/Q_B
$V_1Cr_5O_{11}$/Pt	-1.2	2.12, 1.83<br>1.69,1.72,1.69,1.69	0.1, 2.8<br>-2.7, -2.7, 2.7, 2.7	+0.90	$V^{5+}Cr^{3+}$<br>$Cr^{3+}Cr^{3+}Cr^{3+}Cr^{3+}$
$V_1Cr_5O_{11}$/Pt (inv)	-0.2	2.15, 1.85<br>1.69,1.71,1.69,1.69	0.8, 2.3<br>-2.7, -2.7, 2.7, 2.7	+0.86	$V^{4+}Cr^{4+}$<br>$Cr^{3+}Cr^{3+}Cr^{3+}Cr^{3+}$
$Ti_1Cr_5O_{11}$/Pt	+0.5	2.12, 1.98<br>1.69,1.71,1.69,1.69	0.1, 2.0<br>-2.7, -2.7, 2.7, 2.7	+0.90	$Ti^{4+}Cr^{4+}$<br>$Cr^{3+}Cr^{3+}Cr^{3+}Cr^{3+}$
$Ti_1Cr_5O_{11}$/Pt (inv)	-0.1	2.19, 1.89<br>1.69,1.72,1.69,1.69	0.1, 1.9<br>-2.7, -2.7, 2.7, 2.7	+0.90	$Ti^{4+}Cr^{4+}$<br>$Cr^{3+}Cr^{3+}Cr^{3+}Cr^{3+}$
$V_1Mn_5O_{11}$/Pt	-1.8	2.07, 1.60<br>1.76,1.67,1.68,1.67	0.2, 4.4<br>-3.1, -3.6, 3.7, 3.7	+0.83	$V^{5+}Mn^{2.5+}$<br>$Mn^{4+}Mn^{3.5+}Mn^{3.5+}Mn^{3.5+}$
$V_1Mn_5O_{11}$/Pt (inv)	-1.5	2.10, 1.54<br>1.77,1.67,1.67,1.69	0.2, 4.5<br>-3.0, -3.6, 3.7, 3.5	+0.82	$V^{5+}Mn^{2.5+}$<br>$Mn^{4+}Mn^{3.5+}Mn^{3.5+}Mn^{3.5+}$

Table S3: Calculated substitution energies and electronic properties of VCr_5O_{11}/Pt, $TiCr_5O_{11}$/Pt, and VMn_5O_{11}/Pt nano-spinels with the direct $A^{t}B^{o}O_3$ and the inversed $A^{o}B^{t}O_3$ configuration in the surface layer: Bader cation charges q_A/q_B (e) and magnetic moments μ_A/μ_B (μ_B), Pt Bader charge q_{Pt} (e/cell) and approximate formal oxidation states of cations. For each configuration, results for surface and interface cations in the double-stacks are given in top and bottom lines, respectively. Substitution energies E_{sub} (eV/A) of the mixed AB_5O_{11}/Pt bi-stacks were evaluated with respect to pure B_6O_{11}/Pt films (B = Cr, Mn) and AO_x bulks (TiO_2 and VO_2) at the oxygen condition used in the experiment, $\Delta\mu_O$ = -1.4 eV.

[1] Wang, Y.; Wemhoff, P.I.; Lewandowski, M.; Nilius, N., Electron stimulated desorption of vanadyl-groups from vanadium oxide thin films on Ru(0001) probed with STM, *Phys. Chem. Chem. Phys*. **2021,** *23*, 8439-8445.

[2] Missaoui, G.; Wemhoff, P. I.; Noguera, C.; Goniakowski, J.; Nilius, N., Chromium oxide thin films on Pt(111): An STM and DFT excursion through the phase diagram. *J. Phys. Chem*. C **2024,** *128,* 12726-12734.

[3] Goniakowski, J.; Noguera, C., Properties of metal-supported oxide honeycomb monolayers: M_2O_3 and $MM'O_3$ on Me(111) (M, M′ = Ti, V, Cr, Fe; Me = Ag, Au, Pt). *J. Phys. Chem*. C **2020,** *124,* 8186–8197.

[4] Goniakowski, J.; Noguera, C., Intrinsic properties of pure and mixed monolayer oxides in the honeycomb structure: M_2O_3 and $MM'O_3$ (M, M′ = Ti, V, Cr, Fe). *J. Phys. Chem*. C **2019,** *123*, 7898–7910.
